# A Brief Commentary on the Consensus Definition of Misophonia

**DOI:** 10.3389/fnins.2022.879070

**Published:** 2022-07-07

**Authors:** Jennifer J. Brout

**Affiliations:** International Misophonia Research Network, New York, NY, United States

**Keywords:** misophonia, misophonia etiology, misophonia triggers, auditory-visual perception, misophonia prevalence

## Introduction

A small body of academic literature on misophonia has developed over the last decade. This research includes case studies, experimental studies with small convenience samples, and those with more stringent study designs. For a list of studies see Brout et al. (2018). New hypotheses use prior papers as their basis and therefore some early misconceptions about misophonia and its definition are carried throughout some present-day research across the disciplines of audiology, otolaryngology, psychology, and psychiatry. The result is confusion amongst misophonia sufferers, as well as disagreement across clinicians and researchers. Based on these studies, numerous individuals with misophonia have received treatments that ranged from ineffective to highly uncomfortable. Thus, the need for a consensus definition is clear.

The Misophonia Research Foundation, in partnership with the Center for Strategic Philanthropy, responded to this need by using a modified Delphi method, which structures group communication to deal with a complex problem (Stone Fish and Busby, 2005). It is a lengthy process that begins with the grouping of 15 experts. Experts were comprised of psychologists/psychiatrists, audiologists/ear, nose and throat (ENT) physicians/hearing scientists, a small number of neuroscientists, and one pediatrician. The result of the modified Delphi process yielded a consensus definition that was descriptive in nature, but missed some issues crucial to the misophonia sufferer, omitted some important related literature, and included some contradictory statements.

## Summary of the Consensus Definition

The definition includes a general description of misophonia stating that the disorder is related to a decreased tolerance for pattern-based and repetitive sounds, regardless of loudness. The authors explain that triggers have specific meaning to people and are most often sounds (or related stimuli) emanating from other human beings. The authors suggest that reactions to triggers are mediated by context, the relationship between the individual with misophonia and the trigger source, and the degree to which the one perceives control over the situation. This is a potential contradiction to be discussed in the next section. According to the definition, once an individual with misophonia notices a trigger, they are unable to “distract themselves” from that stimulus. The authors continue to state that misophonia appears to vary from mild to severe, and may impact social, academic, and occupational functioning. Finally, according to the consensus definition, misophonia typically begins in childhood and adolescence. For a thorough version of the consensus definition, please see Swedo et al. (2021).

### Issues to Consider Regarding the Consensus Definition

One statement within the definition stands out as highly contradictory. The authors state that “Misophonic responses do not seem to be elicited by the loudness of auditory stimuli but rather by the specific pattern or meaning to an individual,” while also asserting that “sounds associated with oral functions are among the most often reported misophonic trigger stimuli, such as chewing, eating, smacking lips, slurping, coughing, throat clearing and swallowing” (Swedo et al., 2021). How can most people with misophonia be triggered by the same sounds if these sounds are personal to them?

## The Nature of Sounds and Gating

Attention to the acoustic nature of trigger sounds and how these sounds are neurologically processed may help to parse out this very salient feature of misophonia. However, the papers used in the Delphi study left out this issue and relevant cross-disciplinary work. For example, numerous populations have shown the relationship of sympathetic nervous system arousal in response to repetitive auditory stimuli. In some of these populations, clinicians and researchers commonly suggest co-occurring disorders regarding misophonia. Specifically, studies of children with general sensory processing disorder (SPD) and autism spectrum disorder (ASD) show deficiencies in auditory gating (Brout et al., 2018). Auditory gating is the brain's capacity to selectively regulate sensitivity to auditory sensory stimuli, and is measured through event-related potentials (ERPs) comprised of the P50, N1, and P2 peaks (Brout et al., 2018). Similarly, Schröder et al. (2014) demonstrated that the magnitude of the N1 peak in persons with misophonia was smaller than that in controls, suggesting the possibility of sound encoding deficits in misophonia (Brout et al., 2018). Yet, research consistently demonstrating abnormal sensory processing, measured in terms of early ERP components in the sensory cortex, was overlooked in the consensus definition (Brown et al., 2001; Kisley et al., 2004; Davies and Gavin, 2007; Perry et al., 2007; Jeste and Nelson, 2009; Yadon et al., 2009; Brett-Green et al., 2010; Gavin et al., 2011; Schröder et al., 2014). In addition, when one considers that many individuals with misophonia also report visual sensitivity to movement (Green and Ben-Sasson, 2010) it is appropriate to have considered studies related to visual and auditory cross-modal processes. For further explanation of this see Brout et al., 2018).

These oversights may reflect the authors' collective conclusion that certain language should be left out of the definition of misophonia. The authors state that they cannot commit to any classification of the disorder but suggest that there may be some “underlying organic component.” The committee concluded that “*postulated* mechanisms don't belong in the definition at this time.” Unfortunately, that leaves us with a definition that excludes what is likely the most objective and rigorous research in misophonia, work from the field of neuroscience.

## Considerations Regarding a Dimensional Definition

The consensus definition, then, follows a model of only using observable behavior to identify and classify mental functions. This paradigm, long utilized by the American Psychiatric Association (Schröder et al., 2013) in the DSM (Diagnostic and Statistical Manual) is limited and has been challenged by a more dimensional approach to defining complex mental phenomena. An example of this is the Research Domain Criteria Matrix (RDoC), an initiative of the United States National Institute of Mental Health (American Psychiatric Association, 2013). This movement toward a more dimensional approach to nosology in psychiatry and psychology, allows for the inclusion of perspectives from related fields. As misophonia is a multidisciplinary disorder, a more dimensional definition may be better.

The RDoC framework is a research strategy that involves a matrix of interacting elements related to six major domains of human functions. These domains include sensorimotor systems, arousal/regulatory systems, systems for social processing, cognitive systems, positive valence systems, and negative valence systems. Contained within each of these domains are constructs, or behavioral elements, indicating a range of functioning from typical to atypical. These constructs reflect both neurodevelopmental processes, as well as changes in functioning that may result from environmental influences. For example, the constructs utilize units of analysis related to genetic, neurocircuitry, behavioral, and self-report measures.

The RDoC matrix is designed to evolve along with novel research and strives to provide information about the “basic biological and cognitive processes that lead to mental health and illness.” (National Institute of Mental Health, n.d.). While the RDoC is not yet a diagnostic system, its purpose is to help reconceptualize mental disorders and diagnosis. It seeks to inform mental health measurement, diagnosis, and treatment while increasing knowledge regarding how biological, physiological, and behavioral mechanisms interact (National Institute of Mental Health, n.d.).

## Discussion

The consensus definition is based on a review of research papers that include many, although not all, of these dimensions. For example, the consensus definition is impacted by the absence of rigorous research within certain RDoC constructs, such as genetics. Also, leaving out “*postulated* mechanisms” from the final consensus definition constrains it to mainly observable behavior. Both RDoC and the misophonia consensus definition were designed to evolve as research develops. Therefore, considering a synchronous approach might better serve to explore of all the interacting dimensions of misophonia.

Adding in the “*postulated* mechanisms” to the consensus definition would accomplish this, or at least start the process of this multidimensional exploration. For example, Brout et al. (2018) weaves the six RDoC domains into the definition of misophonia and further explains underlying mechanisms contributing to this disorder. Specifically, this review, along with reports of behavioral observation, self-reports, and initial case studies, includes discussion of central arousal systems and how they relate to the physiological data in misophonia. In a separate section, the paper addresses sensorimotor mechanisms and how these are involved in development and the onset of misophonia, which complies with RDoC standards to include a neurodevelopmental dimension. This dimension also allows for relevant studies related to auditory/sensory gating, which is important given that developmental studies of children diagnosed with atypical sensory processing similar to that in misophonia (e.g., SPD and ASD) tend to demonstrate commonalities in auditory gating deficits (Kisley et al., 2004; Brett-Green et al., 2010; Gavin et al., 2011).

Finally, the consensus definition's well-intended effort falls short in other ways. While it is purported to be a modifiable definition, given the use of the term “expert” it also carries a lot of weight in its original form. It is important to consider that it may be counter-intuitive to call this an expert definition when, by nature of its novelty in research, the underlying mechanisms of misophonia are still poorly understood. Certainly, there is a need for a consensus definition, yet this one reflects the known circularity of the methodology and brings in assumptions from the early literature into the definition. It is then reiterative of the very problems the authors sought to address. Revisiting this definition with a more dimensional approach would be a prudent consideration.

## Author Contributions

The author confirms being the sole contributor of this work and has approved it for publication.

## Conflict of Interest

The author declares that the research was conducted in the absence of any commercial or financial relationships that could be construed as a potential conflict of interest.

## Publisher's Note

All claims expressed in this article are solely those of the authors and do not necessarily represent those of their affiliated organizations, or those of the publisher, the editors and the reviewers. Any product that may be evaluated in this article, or claim that may be made by its manufacturer, is not guaranteed or endorsed by the publisher.
